# Supplementary material for: Integrative CRISPR Activation and Small Molecule Inhibitor Screening for lncRNA Mediating BRAF Inhibitor Resistance in Melanoma
Source: Biomedicines. 2023 Jul 21;11(7):2054. doi: 10.3390/biomedicines11072054 (PMC10377043; doi:10.3390/biomedicines11072054)
Supplement: Supplementary file 1 [file biomedicines-11-02054-s001.zip › Supplementary Figures.pdf]

Figure S1

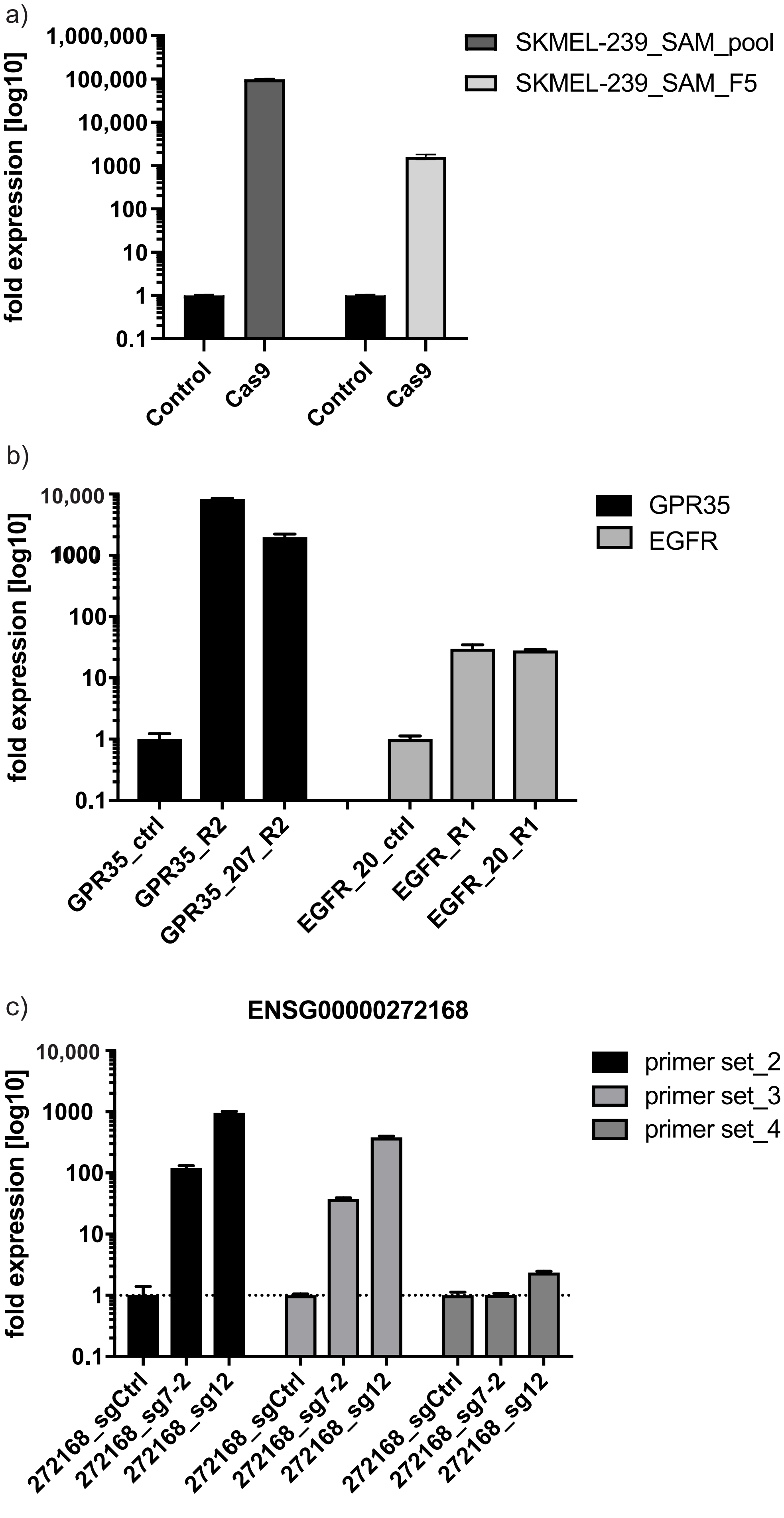

Figure S1. Functional assessment of CRISPRa system in SKMEL-239_SAM_pool and F5 by qPCR expression analysis of Cas9 (**a**), EGFR and GPR35 (**b**) and a randomly expressed lncRNA ENSG00000272168 (**c**).

Figure S2

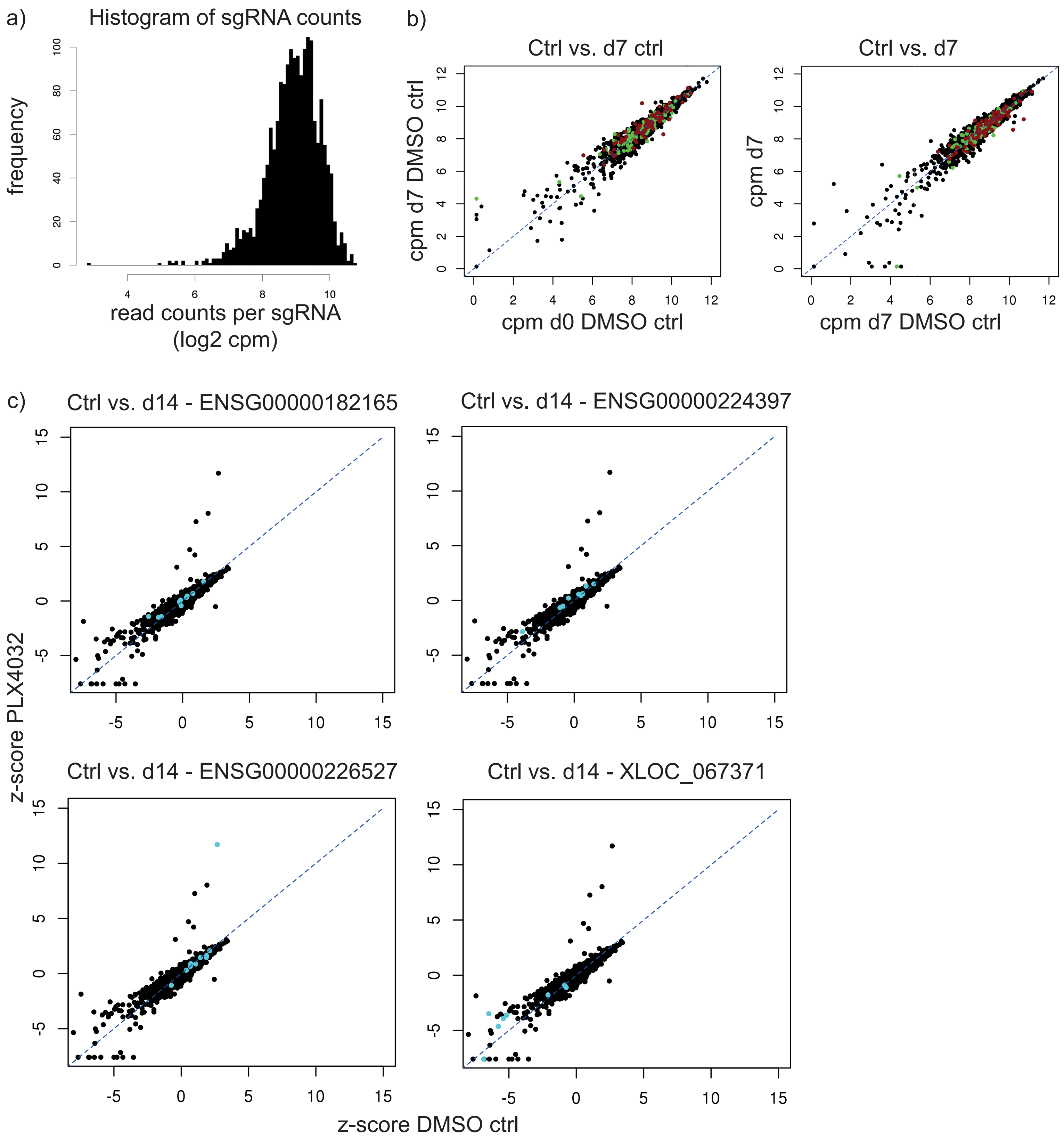

Figure S2. (**a**) CRISPRa sgRNA plasmid library validation. Histogram shows a log2 read counts in cpm against frequency. (**b**) control sample validation at day 7 of (left) cpm of day 0 of DMSO control versus cpm at day 7 DMSO control, (right) cpm of day 0 of DMSO control versus cpm at day 7. (**c**) sgRNA representation of all ten sgRNAs per all four validated lncRNA candidates ENSG00000185168 (aka LINC00482), ENSG00000224397 (aka PELATON), XLOC067371, ENSG00000226527 referring BRAFi resistance in melanoma shown as z-score normalization of DMSO negative control against PLX4032 treated cells at day 14.

Figure S3

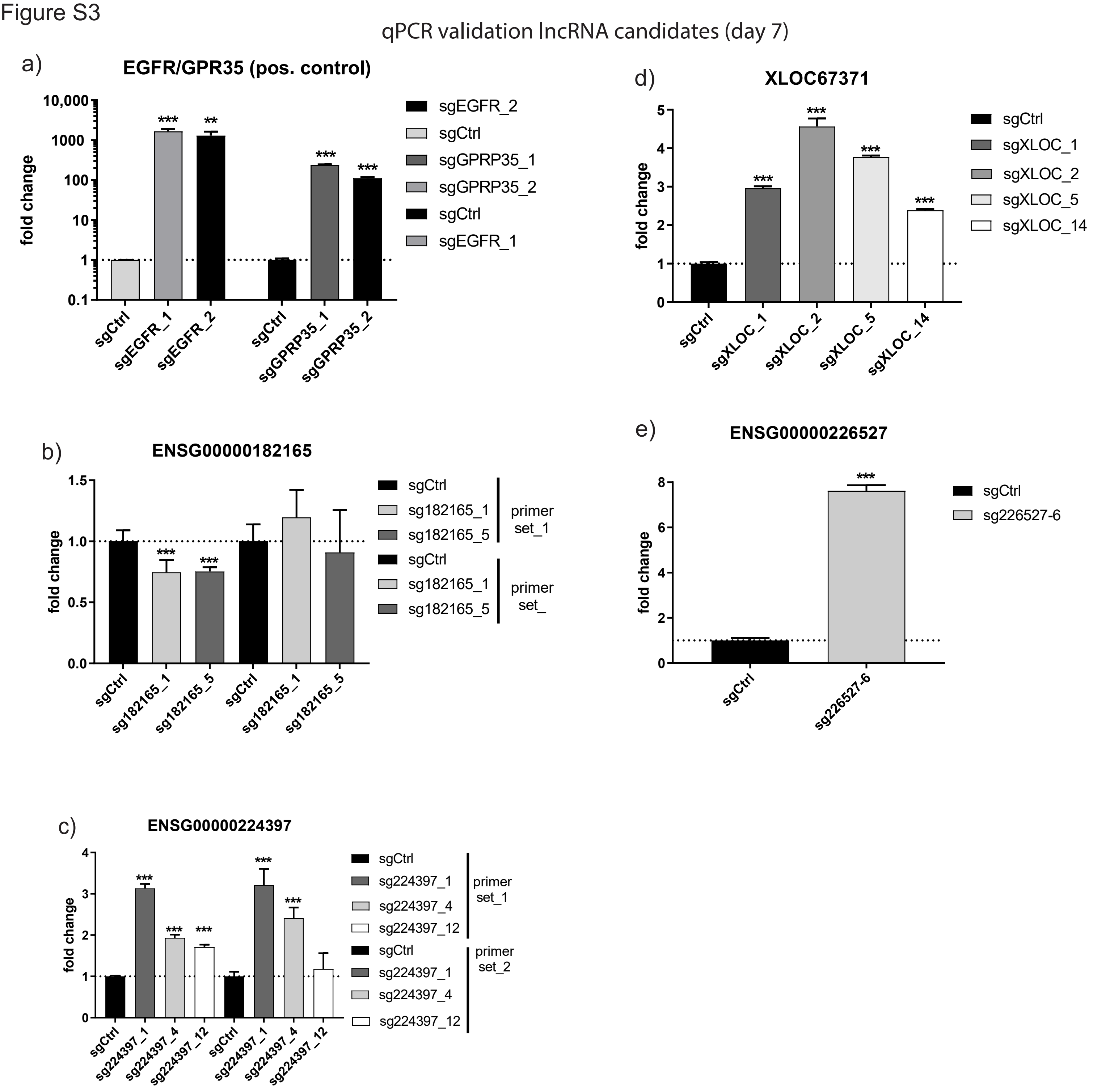

Figure S3. qPCR expression validation of all lncRNA candidates and EGFR positive control after 7 days using diverse primer sets for some lncRNAs.
** $p < 0.01$, *** $p < 0.001$.

Figure S4. IGV genome browser tracks representing one replicate out of three for RNA-sequencing, H3K27ac and H3K4me3 ChIP-Seq for validated lncRNA candidates ENSG00000185168 (**a**), ENSG00000224397 (**b**), XLOC067371 (**c**), ENSG00000226527 (**d**) referring BRAFi resistance in melanoma which are expressed in SKMEL-239 parental, T22 and T23. RefSeq and new lncRNA as well as sgRNAs are shown as blue bars at the bottom.

Figure S4

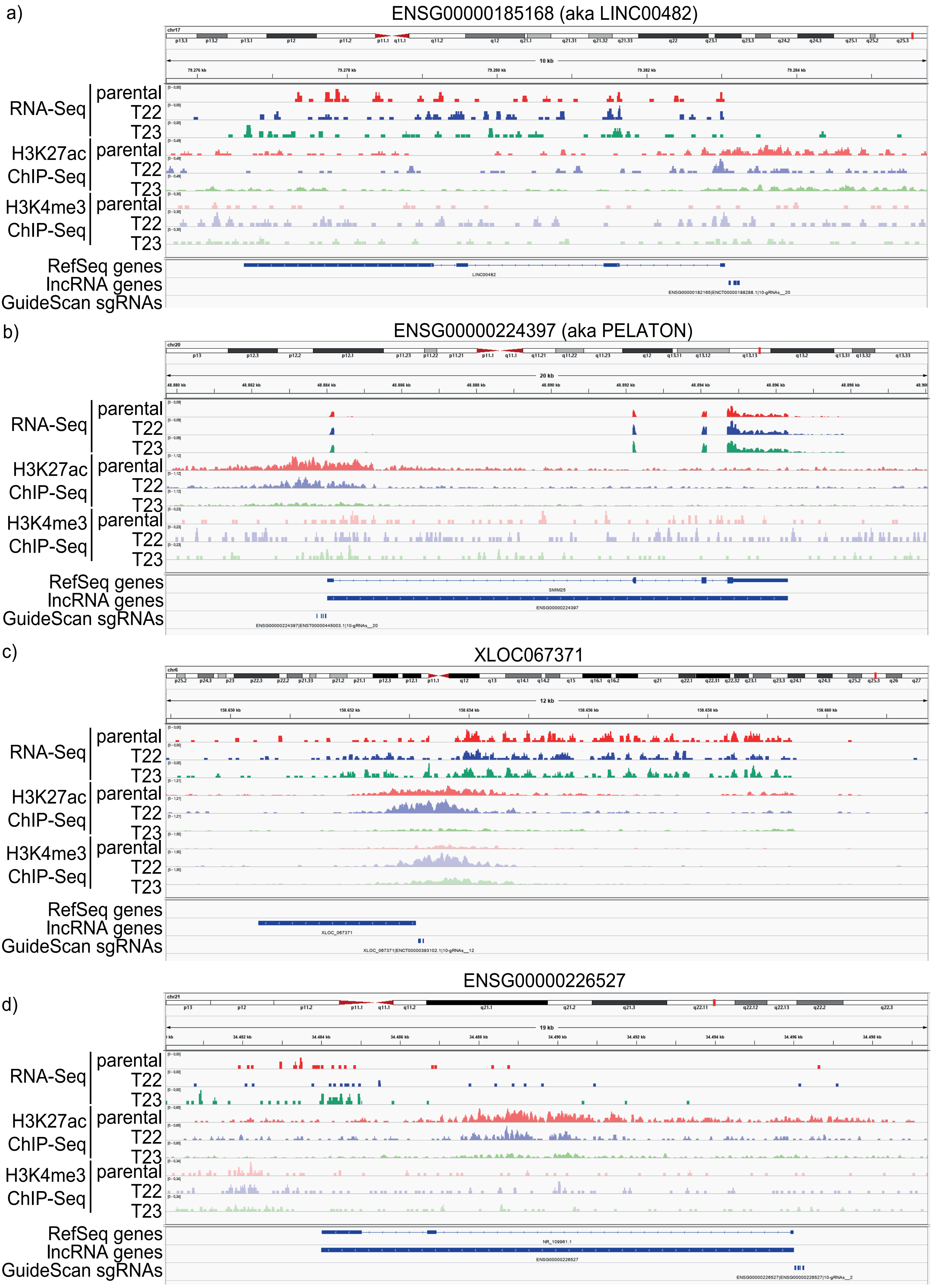

a)
ENSG00000185168 (aka LINC00482)
RNA-Seq
parental
T22
T23
H3K27ac
ChIP-Seq
parental
T22
T23
H3K4me3
ChIP-Seq
parental
T22
T23
RefSeq genes
lncRNA genes
GuideScan sgRNAs
b)
ENSG00000224397 (aka PELATON)
RNA-Seq
parental
T22
T23
H3K27ac
ChIP-Seq
parental
T22
T23
H3K4me3
ChIP-Seq
parental
T22
T23
RefSeq genes
lncRNA genes
GuideScan sgRNAs
c)
XLOC067371
RNA-Seq
parental
T22
T23
H3K27ac
ChIP-Seq
parental
T22
T23
H3K4me3
ChIP-Seq
parental
T22
T23
RefSeq genes
lncRNA genes
GuideScan sgRNAs
d)
ENSG00000226527
RNA-Seq
parental
T22
T23
H3K27ac
ChIP-Seq
parental
T22
T23
H3K4me3
ChIP-Seq
parental
T22
T23
RefSeq genes
lncRNA genes
GuideScan sgRNAs
