# Supplementary material for: Integrative CRISPR Activation and Small Molecule Inhibitor Screening for lncRNA Mediating BRAF Inhibitor Resistance in Melanoma
Source: Biomedicines. 2023 Jul 21;11(7):2054. doi: 10.3390/biomedicines11072054 (PMC10377043; doi:10.3390/biomedicines11072054)
Supplement: Supplementary file 1 [file biomedicines-11-02054-s001.zip › XLOC_067371_Seq.pdf]

>XLOC_067371|chr6:158650472-158653116
CAGCACTGTGAGGGAGATATTGTTTTTGAATGTATGGCTAAAGACACAGCATTTGGAATATTGTTCTTG
TTTGTCACTTGTTAGTAAGTGGTAGAACCAGGACTTCAGTGAAGATATTTGCTCCAAAGACATTCGCAAA
ACCACTGACACTGTGCAAAATGACACAGGCAGAAAACAAACTTGAGGGACTTCTCAGTTTTGCTGAGAG
TTGGGTGTATTTGTACCCAAGAGTGCTGGATAAGAGAGAGTTTATACCTGTGGGCCTTATGGAAAACTA
TGGTATTATTAGAAGAGTAGTTTCAAGGTTTGTATTTTTTTTTTCATTTTGCAGAAGCAAGAACACCTGTA
TCATTCAGTCTTGATGGTATTTGTTGTATCATTTTTATACTGTTACATAGATTGTATTTATTAAGTCAAGAA
AGTTCATCTTTAATCCTTCTTCTCTTTAGTCTGACAGCAAGGGTCAAAATTTCCATAATTTGCTCCAGGCCA
GGGCCAAGCAAATGAATGCTGATCCCTGATCCGTGTGTGTGTGCGCGCGCGCGCAAGAGAGAAAGGGT
TTAAAAAATGTTATGTATATATTTTGGCCAGGAGTGGTGACTCATGCCTGTAATTCCAGCACTTTGGGAG
GCCGAGGTGGGCAGATAACCTGAGGTCAGGAGTTTGAGACCAGCCTGGCCAACATGGTGAAACCTCGT
ATCTATTAAAAATACAAAATTAGATGGGCATGGTGGTAGCGCCTGTAATCCCAGCAATCCCAGCTACTCG
GGAGGCTGAGGCAGGAGAATTGCTTGAACCTGGGAGGCGGAGGTTGCAGTTAGCGGAGATCACACCA
CTGCACCCCAGCCTGGGCAACAGAGTGAGACTCCGTCTCAAAAAAACAAACAAAAAAGTTATGCACACA
TTTTAAAGTAATTCTGTACAGAAATTGTAAATTTTGAAGGCAAGGGCCATATCATATTCTTCCTTTGATCT
CTCCACAGTATTTAACATAACATTGAGCACTAAACTGAAGTGCAATACTAATTACATTGAGCGAAGATTA
TCTTTTAAGGTACTCTAAGATGTAAATGTAGTTTTATTTCTTAAGGTTTTCAAAATTAAGTTTTGGAGTGC
TTATAAAATATAATCAAATAATGCTGATCTTAATGCTTCTATTAAATGCGTTATACATATATATTATATATA
TACTCTTTTTGAGACAGGGTCTCACTCTGTCACCCAGGCTGGAGTGCAGTGGCGCAATCTCGGCTCCCTG
CAACCTCTGCCTCTCGGGGTTCAAAGGATTCTCGTGCCTCAGCCTCCGGAGTAGCTGGGACCACAGTTAA
ATGCATTATATTTATACGCATTCAAACATGAGGCAACTATTCTTGGTTCAGTTACTTTTAAATTAATATTTA
ATTTTCTTGTGTTAGAGCCGGGTTTCGCTATGCTCAGGCTGGAGTCCACAGGCATGATTATAGCTCACTG
CGGCCCCCAGCTCCTGGTCTCCAGGGATCCACCCGCCTCAGCCTCCGGAGTAGCTGGGGACTACACGCC
ACGCTTGGCTTACCTTTTTAAAAATAAAAAAAGTCTAGGTAGCATTTGTCAAAATTTGTAAAATTAGGTC
AAGTGTGGTGGCTCATGCCTGTAATCTCAACACTTTGGAAGGCAGAGGCAGGAGGATCACTTGGAGCC
AGGAGTTCGAGATGAGCCAGGGCAACAGAGCGAAACTCCTACACTGAAAAAAAAAAAATCACAAATGA
GCTGGGCATGGTGGCGGGCACCTGTAGTCCCTAGCTACTCGGGAGGCTGAGGCGAGTATTCATGGAAT
TTCAGAATTTAACAAACTCGGTCCAACTTTTTTTTTGACAAAATTCACAGAATCAGTAATTCTTAAGTCTG
AAACGGATTCGAAAACTTTTGCAGACAGGCAAAACAGAAAACAACGCTGAGCCCTTGCGGAAGAGTCG
CTGCTATTCCTAACTATATGGGGCCTCTAATGCTTCAAATCAAGGAAAAAAGAATGCTTTTTTTTTTAAAC
TACACGTGTGAAGAGTCACACAATCAGCTATGCTATTTCTTTGCACAAGCTGGAAAAAGAAGAGATTAT
CAAGTTTCGTTGTTAGCAGGACATCCATCAAATTTAACAGGAGGGGGAAGGCTGTGAATTGTTCTTCCCT
GAGTTAAAATGACGCTATCCGGTCCTTTCTCCATGGACAACTGAAGAAGAGATCCTCTGGGATGTTACCA
TCGCGGCCACTCGGGTGACCGTTTTCCCGGGGAGCTGCGCGGGTCGGTGGTGACAGCGCTTCTGTGCTC
CCGGCGGCTTTCCCTGTAAGACATAATGGGACTGGGACGTCCACTTCCGGCTCAAACGAAGTTCAGACG
GCGCGCAAGCCGCGCGGTGCCCGGGTGCTGAGGCCGTCCCGAGCCAGGAGACTGGAGGCCTAGGCCC
GGCACTGTGGGCATCTTGGGCACCGTGGCGCGGTGCGGGCCGGGGGCGGGGGTCTCCCGGGGTGGCG
ACGCCGGGGGTGCGAGCAGGTGGCCCGGGCAGGCGGCGGCTCCGGTGGCGGGCGGCGCGGCGGCCC
GCGGGGTGGCGTTGGGGACGCGAGCCCTGGGCCGGGGAGGGAGGGCGGCCGGGGGGCGCCAACGG
CCGGCGGTG
